# Rural Americans’ COVID-19 Vaccine Perceptions and Willingness to Vaccinate against COVID-19 with Their Community Pharmacists: An Exploratory Study

**DOI:** 10.3390/vaccines11010171

**Published:** 2023-01-13

**Authors:** Alexis M. Koskan, Iris E. LoCoco, Casey L. Daniel, Benjamin S. Teeter

**Affiliations:** 1 College of Health Solutions, Arizona State University, 425 N 5th Street, Phoenix, AZ 85004, USA; 2 Mel and Enid Zuckerman College of Public Health, University of Arizona, 550 E Van Buren Street, Phoenix, AZ 85006, USA; 3 Department of Family Medicine, Whiddon College of Medicine, University of South Alabama, 5795 USA North Drive, Mobile, AL 36608, USA; 4 Department of Pharmacy Practice, University of Arkansas for Medical Sciences, 4301 W Markham St., Little Rock, AR 72205, USA

**Keywords:** COVID-19 vaccine, rural, vaccine hesitancy, vaccine willingness, access to care, pharmacy, pharmacy-administered vaccination, immunizations, qualitative research

## Abstract

In early 2022 in the U.S., rural adults were the least likely to vaccinate against COVID-19 due to vaccine hesitancy and reduced healthcare access. This study explored the factors influencing rural adults’ COVID-19 vaccine perceptions and their acceptance of pharmacist-administered vaccination. We utilized phone-based semi-structured interviews with 30 adults living in rural regions of one southwestern state and analyzed the data using a team-based thematic analysis approach. Vaccine-willing participants described knowing other people affected by the virus and their desired protection from the virus. They reported trusting scientific institutions and the government to provide safe vaccines. Vaccine-hesitant populations, however, feared that the COVID-19 vaccine development process had been rushed, compromising the safety of these newer vaccines. Although they differed in the news sources they preferred for receiving COVID-19 vaccine information, both vaccine-willing and vaccine-hesitant participants described trusting local authorities, such as healthcare providers and county government officials, to provide accurate COVID-19 vaccine information. Regarding the acceptability of pharmacist-administered COVID-19 vaccinations, all but one participant described their acceptance of this healthcare delivery approach. Future outreach should leverage rural adults’ trust in local sources, including community pharmacists, deemed more convenient access points to healthcare, when addressing vaccine hesitancy.

## 1. Introduction

In 2021, COVID-19 was the U.S.’s third leading cause of mortality [1]. COVID-19 vaccines offer critical protection against mortality and severe illness due to complications with the virus [2]. Although COVID-19 vaccines have been available tools against the spread of the pandemic, the urban/rural COVID-19 vaccination disparity more than doubled between April 2021 and January 2022 due to vaccine hesitancy and reduced vaccine access [3]. Together, this led to twice as many rural adults dying from COVID-19 compared to their urban counterparts [4]. Between December 2020 to January 2022, rural Americans disproportionately expressed COVID-19 vaccine hesitancy, the refusal or delayed uptake of a vaccine even if it was accessible [5]. Vaccine hesitancy is influenced by individuals’ (1) confidence in the safety and effectiveness of the vaccine; (2) complacency or the low perceived need to vaccinate; (3) convenience in obtaining the vaccine (potential barriers, access to the vaccine); (4) communication of information and misinformation about the vaccine; and (5) context of vaccine acceptance and hesitancy (e.g., the influence of social determinants of health on vaccine perceptions, trust in healthcare providers) [6]. It is critical to explore rural adults’ perceptions of COVID-19 vaccines, including vaccine acceptance and hesitancy, to learn how best to promote future COVID-19 vaccination (booster doses) and vaccination for future outbreaks of other illnesses.

In terms of vaccine access, urban/rural disparities in vaccine uptake were attributed, in part, to vaccine access challenges [7]. A potential solution to reduced vaccine access is to partner with local community pharmacies to disseminate these life-saving vaccines. In rural regions, pharmacies are often more accessible than primary care clinics due to their geographic locations, hours of operation, and lack of required appointments [8,9]. Furthermore, over 75% of rural counties have at least two pharmacies capable of delivering COVID-19 vaccines [10]. However, little is known about rural adults’ willingness to receive pharmacist-administered COVID-19 vaccines. Therefore, this qualitative study explored rural adults’ COVID-19 vaccine perspectives and their willingness to receive such vaccines from their community pharmacist.

## 2. Materials and Methods

This study received Institutional Review Board approval (STUDY00014630). The team utilized purposive sampling to recruit participants. First, they posted study information on various rural towns’ Facebook community pages. However, this recruitment strategy only yielded two participants. After subsequent recruitment efforts (e.g., circulating study fliers to community-based organizations), the team attended a rural community health fair in March 2022, where they recruited most participants. Twelve potential participants provided their contact information and selected a date and time to participate in a one-time phone-based interview.

Guided by the five Cs of vaccine hesitancy (confidence, complacency, convenience, communication, and context) [6], the first author created a draft in-depth interview guide. She tested the interview instrument with two rural adults and updated the guide based on their feedback. See Table 1 for the semi-structured interview guide used in this research.

The study team also administered a demographic survey to assess participant information: biological sex, age, race/ethnicity, work status, income, education, political ideology, and COVID-19 vaccine information (number of COVID-19 vaccine doses received, which vaccine(s) was/were received).

The first author used Google Voice to text participants the day before every interview, reminding them of their scheduled interview. On the day of the interview, she called each study participant, administered the informed consent and demographic survey, conducted the interview, and sent each participant a USD 30 e-gift card after they completed the interview. On average, interviews lasted 21 min and 45 s. A professional transcription company transcribed the audio-recorded interview MP3 files into Word documents. The first author read through the transcribed interviews and continued conducting semi-structured interviews until the study reached data saturation, when no new themes emerged from the data [11].

The team analyzed all demographic data using frequencies. For qualitative data, the team used an inductive thematic analysis approach [12]. The first author read through all interview transcripts and developed a coding guide, listing the names and descriptions of all potential codes that emerged from the data [12]. The first and second authors then coded one article together to test the coding guide and ensure coding similarities. They separately coded three transcripts and met to review their coding outcomes, resolve any discrepancies in how they coded interview data, and update the coding guide as needed [13]. They coded one additional interview separately and later met to ensure coding similarities before they divided the remainder of the uncoded transcripts to be analyzed and coded independently. Throughout the individual coding process, the authors alerted each other whenever a new theme emerged so they could incorporate new codes and recode materials they previously analyzed. The first author entered the coded materials into ATLAS.ti software (ATLAS.ti Scientific Software Development GmbH, Berlin, Germany), and used the software to organize quotes by theme. Finally, they created summaries of each theme and identified quotes representative of participants’ perceptions.

## 3. Results

A total of 30 participants completed interviews. Of the participants, the majority (n = 23, 76.7%) were willing to receive COVID-19 vaccines (“vaccine-willing”), and all but one (n = 22, 73.3%) received the original COVID-19 doses and at least one booster dose. The one vaccine-willing participant who had not received any COVID-19 vaccine doses reported previous severe reactions to common vaccines and provider recommendation to forgo vaccinating against this virus. The remaining participants were “vaccine-hesitant,” and they reported either being completely resistant to vaccinating against COVID-19 (n = 4, 13.3%) or had become more vaccine-hesitant over time and made a conscious decision to forgo receiving additional doses (n = 3, 10%). See Table 2 for the demographic characteristics of participants, organized by their vaccination status.

For the qualitative data, we present our findings from COVID-19 vaccine-willing and vaccine-hesitant individuals, comparing their vaccine perceptions, trusted information sources, and acceptance of pharmacist-administered COVID-19 vaccinations.

### 3.1. COVID-19 Vaccine Perceptions

See Table 3 for influences on rural adults’ perceptions of COVID-19 vaccines. Among the participants who expressed willingness to vaccinate against COVID-19, their perceptions were influenced by their perceived susceptibility to becoming infected with COVID-19, their likelihood of experiencing complications, their desired protection against the virus, and their trust in vaccines, science, and the government.

Most vaccine-willing participants described how they knew at least one other person who had died or been hospitalized due to complications with COVID-19, which influenced their vaccine-seeking behaviors. For example, one participant reported being hospitalized for more than one month due to complications with the original COVID-19 strain before the availability of COVID-19 vaccines. To avoid future complications with this virus, she sought the vaccine. Another participant described a family member who, even after vaccines became available, had not received any COVID-19 vaccine doses and had been hospitalized due to severe infection. Knowing someone affected or simply being in contact with or exposed to someone who became ill with COVID-19 motivated participants to begin the initial recommended course of vaccinations. Participants further described their rationale for seeking COVID-19 vaccines, which predominantly focused on protecting their health and the health of family and friends, including immunocompromised individuals.

Other vaccine-willing participants reported trusting vaccines and science, describing their previous unquestioning nature of receiving other immunizations such as the flu, polio, shingles, and pneumonia vaccines. A few participants shared stories of how, as children, they waited in lines to receive a polio vaccine, recalling how they were administered orally on sugar cubes. Others discussed how immunizations were required for school entry and did not question whether to vaccinate. In addition, they reported their trust and lack of hesitation to receive COVID-19 vaccines due to their confidence in the government to oversee the production and distribution of vaccines that are safe for the public.

Vaccine-hesitant adults described their concerns about COVID-19 vaccine safety and perceived manipulation of COVID-19-related information by the government and the media. Their perceptions of safety were influenced by the rapid speed of COVID-19 mRNA vaccine development, the fear of both short- and long-term side effects of these new vaccines, and the fear that mRNA COVID-19 vaccines could alter individuals’ genetic code. The theme of intentional manipulation of COVID-19 information included opinions that the U.S. government, including state and federal agencies, conspired to overstate the seriousness of the virus, and exaggerated or manipulated data related to the number of COVID-19 deaths and hospitalizations. Furthermore, two participants described the governments’ withholding of COVID-19 treatment efforts, particularly COVID-19 anti-viral medications that could reduce the severity and longevity of the infection’s symptoms.

### 3.2. Trusted and Untrusted COVID-19 Vaccine Sources

We asked participants about their information sources and information-seeking practices to explore what influenced their perceptions about COVID-19 vaccines. See Table 4 for the differing information practices by their willingness to vaccinate against COVID-19.

Vaccine-willing adults trusted numerous sources for receiving health information. For example, many reported watching PBS (Public Broadcasting Service) and local news reporting on county-level health information for the most up-to-date COVID-19 vaccine-related news. Additionally, vaccine-willing adults trusted information disseminated by federal-level medical and public health professionals, often citing their trust in Dr. Anthony Fauci (the Chief Medical Advisor to the President) and the Centers for Disease Control and Prevention. In addition, participants expressed confidence in their medical care providers, including their primary care providers and pharmacists, to share accurate health information. Some described approaching their healthcare professionals with specific COVID-19 questions, trusting that these professionals would not intentionally deliver misleading information. One participant described his trust in his community pharmacist, emphasizing the importance of a healthcare authority who lives and works in the local community.

When asked who they distrusted for COVID-19 vaccine information, vaccine-willing adults cited their lack of trust in national news organizations, specifically Fox News, for COVID-19 vaccine information. They described how these sources spread misinformation and contributed to the politicization of public health and science. They also reported their lack of trust in information shared on social media by non-experts. Many participants described not trusting social media, specifically Facebook, with a few articulating that they believed this is how vaccine misinformation spread rampantly.

Although vaccine-hesitant populations distrusted the media, they reported relying on conservative news media as sources for COVID-19 vaccine information. They were also more likely to describe processes for seeking information and relying on their powers of intuition to identify accurate information and their discernment to make sense of the information they received. In addition, they described their processes for collecting and interpreting health information, mindful of the politicization of health information. The three participants who received at least one COVID-19 vaccine dose but became vaccine-hesitant over time reported reading Robert Kennedy’s book (titled *The Real Anthony Fauci*: *Bill Gates*, *Big Pharma*, *and The Global War on Democracy and Public Health*). Over time, these individuals became more hesitant about receiving additional COVID-19 vaccine doses, especially since the booster doses available were mRNA COVID-19 vaccines.

### 3.3. Acceptability of Pharmacist-Administered Vaccinations

All but one participant (vaccine-hesitant) trusted community pharmacists for health information and to deliver vaccines safely, and half (n = 15) of participants described receiving one or more COVID-19 vaccine doses at their community pharmacy. Participants described numerous reasons for their willingness to receive vaccines from their local community pharmacist. Table 5 illustrates participants’ beliefs about the acceptability of pharmacist-administered vaccinations as a healthcare delivery approach.

Their stated reasons for accepting pharmacist-administered vaccinations included previously receiving immunizations from their pharmacist, their beliefs that pharmacists are trustworthy and knowledgeable healthcare providers, physician recommendation to receive vaccines with their community pharmacists, and the convenience of this healthcare delivery approach. They described convenience in terms of geographic proximity to their homes, the lack of a needed appointment to receive vaccines, and knowing that pharmacies stocked COVID-19 vaccines.

## 4. Discussion

This qualitative study adds to the body of research on COVID-19 by exploring rural American adults’ perspectives about COVID-19 vaccines and the information sources that informed their views about this disease prevention resource. Nationwide, this diverse population has expressed higher rates of vaccine hesitancy than the general U.S. population. However, among this study sample, the majority had received the full recommended course of COVID-19 vaccine doses and boosters (n = 23, 76.7%). These higher vaccine acceptance rates may be due to the participants’ ages. Those fully or partially vaccinated were older adults (over age 65 years), a population who, nationally, has the highest rates of vaccination (95% of older adults are vaccinated) [14]. In the U.S., older adults were prioritized to be among the first to receive COVID-19 vaccines to reduce their risk of hospitalizations and death. The media coverage and government emphasis on prioritizing older adults to receive COVID-19 vaccines may have also influenced their willingness to vaccinate against COVID-19. On the other hand, younger study participants more often expressed COVID-19 vaccine hesitancy, with most unvaccinated respondents belonging to the 50-to-64-year-old age category. Nationally, 50-to-64-year-old rural-living adults report COVID-19 vaccine refusal at a rate of 19%, with an additional 9% stating that they will only accept vaccination if required [15].

Similar to recent research with older adults, vaccine-willing participants recalled previous vaccine rollouts, such as the national campaigns to eliminate smallpox and polio [16]. One meta-analysis on behavior change and risk appraisal demonstrated that the greatest degree of behavioral change occurs when individuals anticipate a high response efficacy of an intervention [17]. Witnessing a previous mass vaccination intervention’s success could influence individuals’ perception that vaccine campaigns help protect communities against diseases. It is plausible that individuals in the 45-to-64-year-old age group may not remember the success of previous mass vaccination campaigns, and therefore lack this perceived vaccine campaign response efficacy. One avenue for future research could examine the role that past personal experiences of previous mass vaccination campaigns, and the success these vaccination campaigns, could play in influencing vaccine attitudes across different age cohorts and generational subgroups.

Among vaccine-willing participants, personal experience of becoming ill with COVID-19 and having friends or family who became sick or died due to complications of COVID-19 greatly influenced their vaccine-seeking behaviors [18]. Previous research found that individuals who experienced COVID-19 infection themselves or experienced the illness or death of a family member due to COVID-19 reported higher levels of COVID-19-related anxiety, and were more likely to adopt preventive behaviors to mitigate perceived risk [19]. On the other hand, vaccine-hesitant participants who believed that the government and media exaggerated COVID-19 hospitalizations and deaths may have negatively impacted their perceived susceptibility and severity of the virus, therefore leading to greater vaccine complacency and hesitancy.

Vaccine hesitancy was a matter of fear, stemming from a lack of trust in vaccine safety and the vaccine development process (confidence in the vaccine), particularly the speed that COVID-19 vaccines were developed, tested, and subsequently deployed. Some perceived that the speed of the vaccine development process sacrificed safety and efficacy to enable rapid public availability [20]. It is important to note that the combination of available mRNA-vaccine-producing technology, a pandemic, and extensive funding from various other nations led to pharmaceutical companies’ abilities to mass test and produce vaccines [21]. This pandemic paradigm for vaccine creation and federal-level financial investment allowed pharmaceutical companies to create and test vaccines without fearing significant financial loss. It is critical to build public trust in the mRNA vaccine development process as these technologies become more ubiquitous in creating new vaccines.

Participants who became more distrustful of COVID-19 vaccines over time believed the government promoted COVID-19 vaccination while withholding effective medications (specifically, the unproven treatments of ivermectin, an anti-parasite medication for animals, and hydroxychloroquine, a drug used to treat malaria that has been disproven to prevent or treat COVID-19). All who voiced these concerns also reported reading the same book written by Robert F. Kennedy, Jr., an anti-vaccine advocate. Previous research that assessed COVID-19 vaccine misinformation posted on Twitter in 2021 reported that Robert F. Kennedy Jr. had among the most retweeted COVID-19 vaccine misinformation messages [22]. In fact, the Facebook and Instagram accounts for the Children’s Health Defense, Kennedy’s non-profit organization, were banned due to repeatedly posting vaccine misinformation [23]. The Center for Countering Digital Hate, a non-profit organization that identifies misinformation and hate speech online, has named Kennedy as part of the Disinformation Dozen, one of twelve individuals responsible for disseminating 65% of anti-vaccination misinformation online. Recent research reported that his organization paid for over 50% of Facebook advertisements with anti-vaccine messages [24,25]. It is critical for the public to learn that this prominent figure is using multiple platforms such as books, YouTube videos, documentaries, and advertisements to spread vaccine messages, that, when fact-checked, are flagged as misrepresentations of science [26].

When asked about sources participants do not trust for COVID-19 information, most described their lack of trust in national-level politicians, including former President Trump, and politically informed news media. Furthermore, participants expressed their disdain for COVID-19 becoming weaponized for political gain. Bolsen and Palm described three ways that the pandemic and COVID-19 vaccines became politicized, which include (1) through the political and media coverage of the COVID-19 vaccine development process, (2) the alignment of vaccine development before a U.S. presidential election, and (3) the international competition to be the first to develop a safe and effective COVID-19 vaccine [27]. The politicization of the pandemic and of science led to increased hesitancy and resistance to vaccinate against COVID-19 [27]. In future vaccine campaigns, it is essential to avoid politicizing disease prevention resources. Emphasizing scientific and medical expert recommendations for safe and effective vaccines can help increase the public’s confidence in these life-saving resources [28].

Vaccine-willing and vaccine-hesitant participants described their trust in COVID-19 information from local agencies, such as county and state health departments, and interpersonal health professional sources, such as primary care providers and pharmacists. Strategies for increasing vaccine uptake and reducing the spread of COVID-19 should capitalize on the trust between rural community members, pharmacists, primary care providers, and local health authorities, and explore avenues of increasing productive dialogue and education about COVID-19. Discussions with healthcare providers, more specifically, may more effectively address and reduce vaccine hesitancy and vaccine skepticism than media campaigns or one-way education pathways [29]. Future research should identify methods of engaging local healthcare providers in partnership with trusted local sources (e.g., local news channels, local government officials, and state and country health departments) to spread accurate, trustworthy information.

Participants in this study trusted pharmacists to provide vaccinations, particularly COVID-19 vaccinations. Past research exploring rural adults’ acceptance of pharmacist-administered immunizations focused on parents’ acceptance of having their adolescent children receive pharmacy-based vaccinations [30,31]. Similar to this study’s findings, previous individuals reported support for pharmacist-administered vaccines due to the convenience of this healthcare delivery approach. They reported the advantage of vaccinating at pharmacies instead of other healthcare clinics for the following reasons: pharmacies are within closer geographic proximity, lack of required appointments to receive most vaccines, hours of operation, and the ability to receive vaccines quickly [6,7]. Individuals who did not want to receive pharmacist-administered COVID-19 vaccinations more often reported a lack of trust in the vaccines, not their pharmacists. Given rural adults’ high levels of trust in pharmacists, future research should explore communication strategies pharmacists can employ to reduce COVID-19 vaccine hesitancy. Past research has suggested that pharmacists confident in recommending vaccines are well-poised to promote vaccination and reduce vaccine hesitancy among their patients with up-to-date, relevant training and information about COVID-19 vaccines [32]. When provided with communication training, pharmacy staff can identify unvaccinated adults and address their vaccine concerns, increasing vaccine uptake [33,34]. Past research reported increasing pharmacist-administered vaccination rates for five vaccines by up to 10% in rural regions when pharmacists were trained to identify vaccine-eligible adults and provide patient counseling about specific vaccine concerns [34]. Another study reported patients’ increased willingness to vaccinate if pharmacists strongly recommended vaccinating [35]. While most research supports presumptive language (presupposing patients want the vaccine) when making vaccination recommendations [36], it is unclear whether this approach effectively addresses vaccine hesitancy in nonmetropolitan adults. Shared clinical decision-making may improve patients’ trust in their healthcare providers (including pharmacists) and address vaccine hesitancy in these rural populations [37]. Future research should test the impact of pharmacy-based patient communication interventions for increasing COVID-19 vaccine uptake among nonmetropolitan adults—populations who may have different communication preferences than their more urban counterparts.

### Limitations

It is important to note several limitations when interpreting the findings from this qualitative study. First, most participants were selected from a single community due to difficulties recruiting rural participants from across the state. The demographic makeup of this community differs from the state and U.S. rural population as a whole, with participants who were, on average, more educated and wealthier than most rural-living adults. This sample was also not especially diverse, with most participants identifying themselves as non-Hispanic Whites. Participants were also older, at higher risk of complications due to COVID-19, and more likely to be of retirement age (73.3% were 65 years or older) compared to the total U.S. rural population (of which 17.5% were over age 65 years according to the most recent census data) [38]. Furthermore, given that participants were predominantly recruited from one rural community, relationships among participants may exist. This may have impacted their communication around COVID-19 and COVID-19 vaccines and in sharing resources (e.g., Kennedy’s book, *The Real Anthony Fauci*) related to the pandemic. Future research should survey a random sample of rural adults for more generalizable findings.

## 5. Conclusions

Vaccinating against COVID-19 is critical to prevent and control the spread of COVID-19. While most rural adult participants surveyed were not vaccine-hesitant, those who showed vaccine hesitancy were those under 64 years old. The most common reasoning for vaccine hesitancy included a lack of trust in vaccine safety and fear of receiving incorrect information about the severity of COVID-19 from the government and media. Vaccine-willing and vaccine-hesitant rural adults were more likely to trust local sources of health information, especially their healthcare providers. Furthermore, in rural regions, pharmacy-administered vaccination, including COVID-19 vaccinations, is an acceptable healthcare delivery approach. Therefore, it is essential to encourage individuals in these less populated regions to seek COVID-19 vaccination at their community pharmacies to increase access and reduce barriers to receiving these vaccines.

## Figures and Tables

**Table 1:** Semi-structured interview guide.

To begin our interview, I would first like to learn your perspectives on COVID-19. In what ways has COVID-19 affected you?
My next questions are about COVID-19 vaccines. Did you vaccinate against COVID-19? If vaccinated What made you decide to get the vaccine? What was your experience getting a COVID-19 vaccine? If unvaccinated What made you avoid getting COVID-19 vaccinations?
My next questions are about sources you trust for COVID-19 vaccine information. Who do you trust to share accurate information about COVID-19 vaccines? Who do you not trust to share accurate information about COVID-19 vaccines? What has been your experience in analyzing information about COVID-19 vaccines? Is there anything else you would like to share about your beliefs about COVID-19 vaccines?
My next set of questions is related to your willingness to receive COVID-19 vaccines and vaccine information from your community pharmacist. How willing are you to receive vaccines from your community pharmacist? How willing are you to get the COVID-19 vaccine with your community pharmacist?
What impacts your willingness to receive COVID-19 vaccines from your pharmacist? What factors would influence your decision to receive a COVID-19 vaccine from your community pharmacist?
What other information would you like to share about COVID-19 and COVID-19 vaccines?
Thank you for taking the time to speak with me about your experiences and perceptions of COVID-19 vaccines. I certainly appreciate your valuable input. This ends our interview.

**Table 2:** Participant demographics by vaccination status.

	Fully	Partially	Not
	Vaccinated	Vaccinated	Vaccinated
	(n = 22)	(n = 3)	(n = 5 *)
Age	73 (SD = 11.32)	77.6 (SD = 11.02)	52.8 (SD = 17.88)
Sex			
Female	16 (72.7%)	1 (33.3%)	3 (60%)
Male	6 (27.3%)	2 (66.6%)	2 (40%)
Race/Ethnicity			
Non-Hispanic White	19 (86.4%)	3 (100%)	4 (80%)
Hispanic White	1 (4.5%)	-	-
Native American	1 (4.5%)	-	-
Black	-	-	1 (20%)
Other	1 (4.5%)	-	-
Education			
Some college	6 (27.3%)	1(33.3%)	1 (20%)
College graduate	7 (31.8%)	2 (66.7%)	1 (20%)
Some postgraduate work	-	-	2 (40%)
Postgraduate degree	9 (40.9%)	-	1 (20%)
Employment			
Full-time	4 (18.2%)	-	2 (40%)
Part-time	1 (4.5%)	-	1 (20%)
Retired	17 (77.3%)	2 (66.7%)	1 (20%)
Unable to work	-	1 (33.3%)	1 (20%)
Income			
USD 0–25,999	2 (9.1%)	1 (33.3%)	1 (25%)
USD 26,000–51,999	4 (%)	-	-
USD 52,000–USD 75,000	1 (4.5%)	-	1 (25%)
Over USD 75,000	9 (40.9%)	2 (66.6%)	3 (50%)
Decline to say	6 (27.3%)	-	-
Political Affiliation			
Liberal	2 (9.1%)	-	1 (20%)
Slightly liberal	5 (22.7%)	-	-
Moderate	6 (27.3%)	1 (33.3%)	-
Slightly conservative	1 (4.5%)	-	-
Conservative	6 (27.3%)	1 (33.3%)	2 (40%)
Extremely conservative	2 (9.1%)	1 (33.3%)	2 (40%)

* This number includes the participant who was willing to vaccinate against COVID-19 but did not vaccinate due to previous severe adverse reactions to vaccines.

**Table 3:** Perceptions of COVID-19 vaccine-hesitant and vaccine-willing adults.

Vaccine-Willing		
Perceived susceptibility to COVID-19	Effects of lack of vaccination	We have a cousin—50 years old—that was very, very sick. She had to be airlifted to the nearest hospital. And I think people never realized that the pandemic is one thing, but to lose a relative, a young relative, how devastating that would have been for all of us. Her doctor had told her that she probably didn’t need the shot because she was healthy. To have lost her because she didn’t have the COVID shot would have been very traumatic in a traumatic time. And I think a lot of people, perhaps, didn’t realize what their death could have meant to everyone around them. (Participant 6)
	Knowing someone who was infected with COVID-19	We used to have a neighbor who used to come around to pick up the paper every day. And so, she did get COVID from some people who decided they were gonna go to a big birthday party in some restaurant when the restaurants opened up. So, that’s when we went for the test and found out we didn’t have it. And so, after that, we took the first shot. (Participant 27)
Protection against COVID-19	Protect self	I was anxious to get it to have some kind of protection. (Participant 11)
	Protect family	What it came down to is I wanted to get more protection for my wife than anything else because she is allergic to so many different things out there. Since we both are out and about, I didn’t want to risk getting it and bringing it home to her and her not doing well with it. Protecting my wife and other family members was my primary reason for getting it. (Participant 18)
Trust	Previous vaccination efforts	You know, I’m old, and I grew up in a time when we lined up to get the polio shot. So, when this COVID shot came out, I didn’t question the vaccine. I just knew we had to get it. (Participant 17)
	Trust in science	I believe in science, and what scientists are saying-and not what some truckers say. (Participant 6)
	Trust the government to create safe vaccines	I do trust the government when it comes to vaccinations. They usually do many studies before they release things. Not necessarily vaccines, but drugs in general. They have double-blinded studies and run them over and over again. (Participant 3)
**Vaccine-Hesitant**		
Vaccine Safety	Rapid vaccine development	I’m not anti-vax. I’m anti-COVID vax… For any other kind of vaccine since the vaccine has been invented—I think in the 1700s or 1800s—the CDC has never released the vaccine for years. Five, six, seven, eight, and ten years… Now we have the CDC telling us the vaccine was safe in a matter of months. (Participant 20)
	Long-term side effects	My major concern is the long-term side effects that I could end up developing. (Participant 2)
	Fear that vaccine changes DNA	And now all the studies come out saying, “Well, it’s really not even mRNA. It’s not a vaccine. It’s gene therapy, so it modifies your body at a cellular level”. And I feel like, man, people were just lying to our faces in order to make us get it. (Participant 7)
Manipulation of COVID-19 information	Misrepresentation of COVID-19 severity	I feel like they are playing games with us. I feel like these people probably had symptoms of some other illness that look identical to COVID. And they (the government) just took advantage of the situation and told us they died of COVID. (Participant 2)

**Table 4:** Information sources and practices used by COVID-19 vaccine-hesitant and vaccine-willing participants.

Vaccine-Willing Adults’ Perceptions	Vaccine-Hesitant Adults’ Perceptions
Trusted health information sources	
Public Broadcasting System (PBS): PBS. It’s the news hour that comes on at 6:00 every night, and we followed it quite carefully; the up-to-date regular information… not so much the national news, you know, NBC or CBS, but the public health, public service news. (Participant 11)	Conservative news stations: I’m very conservative, so I get my news from conservative stations and podcasts. I like Real America’s Voice and Steve Bannon. He’s on five days a week, and usually, you can get documented information. They also have real doctors that you can follow on these shows. They don’t just agree with what the government is telling us. (Participant 14)
Dr. Anthony Fauci: I did listen to Dr. Fauci for quite a while. I thought that his information was as accurate as we could get. I have not changed my mind on that. (Participant 19)	Compiles own information: I generally gather everything I can find. I don’t know. Who can you trust? Where do you look? I get everything from all points of view. Then it’s just a process of elimination. You learn over time about how information sources tilt one way or another. Sometimes you can just tell by reading. It’s pretty obvious. So, then I dig deeper. Maybe from somebody coming at it from a different angle. (Participant 7)
Centers for Disease Control and Prevention: I have to say, I did listen to the CDC. (Participant 16)	Various scientific, non-political sources: I trust what makes sense to trust—medical reviews, epidemiologists working around the world—and who tell you that wearing a mask doesn’t protect you. They don’t have anything to gain. They have no political affiliation. They don’t want to become a star in the government. (Participant 20)
Local government: I trust information coming from my county. My county seems to be on top of everything and gives us what I consider accurate information for our area. I consistently watch the reports—they report it in the news. I also look at stuff on the county website. (Participant 4)	Robert Kennedy’s Book: The book is by a Kennedy. It’s a best seller on Amazon. It is disturbing the things that you find out… Now here’s the one thing that I think is strange. They have medicines you can take (to treat COVID-19). One kind can treat a horse with worms (ivermectin). The other one is hydroxychloroquine. Those pills have been on the market for over 40 or 50 years. They have been used all around the world for different things… no bad results. So, why are they now banned in the United States? You can’t get them. You have to go to a foreign country to get it or have it compounded. Read the first 30 pages of the book. (Participant 26)
Primary care provider: I think whatever comes from the primary doctors is from the clinic because we know they’re not going to manipulate the information. (Participant 29)	-
Pharmacist: Normally, they’re local; they live in the community. And I would tend to, I’m not going to say, befriend, but tend to trust them more than, let’s say, somebody on the opposite end from Washington DC. (Participant 4)	-
Untrusted Health Information Sources	
News media: They literally said masks were useless on CNN. And I used to trust that source. And I don’t trust that source anymore. And MSNBC, I don’t see as a credible source because they are agenda-driven. Fox has its own agenda. So, the place that you used to be able to go for unbiased, factual information doesn’t exist anymore. (Participant 23)	News media: I don’t trust mainstream media. They’re just a propaganda arm. Whatever the liberal socialists want to say is what they say. (Participant 7)
Government: I wasn’t trusting what was coming out of the government because I strongly believe that the different departments were speaking with a forked tongue. One minute it’s “Follow this regulation”. And then somebody else, and then the CDC…. And the messages were just so mixed. You didn’t know who to trust. (Participant 24)	Government: The government… I don’t trust them at all. (Participant 14)
Politicians: I don’t understand these people listen to Trump, you know? And he wanted us to drink Clorox and do all these crazy things, and people listen to him. And a lot of them wouldn’t get shots because of that crazy man, and that’s no way to run a country. We’re supposed to be together. (Participant 25)	-
The information posted on social media: You get a lot of junk on Facebook and other places. A lot of misinformation has been passed through those channels. I don’t look there for anything (any information). (Participant 25)	-
Non-health professionals: My theory is, I don’t have somebody fix my plumbing that isn’t a qualified plumber. And I don’t have somebody do electrical work that isn’t an electrician. So, I’m not going to listen to somebody’s health opinions who don’t work in the field (of health or medicine). (Participant 16)	-

**Table 5:** Participants’ willingness to vaccinate with pharmacists.

Theme	Supporting Quote
Receiving other vaccines from pharmacist	I’ve been getting my vaccines for the last two years through my local pharmacy. My flu vaccine, my shingles vaccines, COVID vaccines, and things like that. (Participant 15, vaccine-willing)
Trust in pharmacist	I have taken my flu shots, pneumonia shots, and shingles shots, and I take them from my pharmacy. I’ve requested them, and I’ve got a good measure of trust in my pharmacy to do what is right. (Participant 9, vaccine-willing)
Trusted health professional	A pharmacist is a very highly trained professional. This is not a second-rate profession. (Participant 3, vaccine-willing)
Physician recommendation	That’s when I talked to my doctor about getting the booster she said the best thing to do is to go to your local pharmacy. (Participant 13, vaccine-willing)
Reduce burden on physician	I don’t want to make an appointment with my primary care physician to take up his time when I can go to the pharmacy and take up their time, I guess. (Participant 7, vaccine-hesitant)
Convenience: location	I like my pharmacist because they’re right around the corner. And you don’t have to make an appointment. (Participant 16, vaccine-willing)
Convenience: no appointment is needed	I don’t have to go to see my doctor, make an appointment, wait five days, six days. So, convenient. It’s convenient. (Participant 20, vaccine-hesitant)
Convenience: access to the vaccine	Yeah, I needed my (COVID-19) booster; they had it. So, I went online and made an appointment. It was really that simple. (Participant 8, vaccine-willing)

## Data Availability

Data will be available upon request.
